# Intravenous Ketamine Bolus(es) for the Treatment of Status Epilepticus, Refractory Status Epilepticus, and Cluster Seizures: A Retrospective Study of 15 Dogs

**DOI:** 10.3389/fvets.2021.547279

**Published:** 2021-02-17

**Authors:** Patrick Roynard, Ann Bilderback, Curtis Wells Dewey

**Affiliations:** ^1^Long Island Veterinary Specialists, Department of Neurology/Neurosurgery, Plainview, NY, United States; ^2^VCA Northwest Veterinary Specialists, Clackamas, OR, United States; ^3^Elemental Pet Vets, Freeville, NY, United States

**Keywords:** ketamine, status epilepticus, refractory status epilepticus, cluster seizures, epilepsy

## Abstract

Status epilepticus (SE) and cluster seizures (CS) are common occurrences in veterinary neurology and frequent reasons of admission to veterinary hospitals. With prolonged seizure activity, gamma amino-butyric acid (GABA) receptors (GABAa receptors) become inactive, leading to a state of pharmacoresistance to benzodiazepines and other GABAergic medications, which is called refractory status epilepticus (RSE). Prolonged seizure activity is also associated with overexpression of *N*-methyl-D-aspartic (NMDA) receptors. Rodent models have shown the efficacy of ketamine (KET) in treating RSE, and its use has been reported in one canine case of RSE. Boluses of KET 5 mg/kg IV have become the preferred treatment for RSE in our hospital. A retrospective study was performed to evaluate and report our experience with KET IV bolus to treat prolonged and/or repeated seizure activity in cases of canine CS, SE, and RSE. A total of 15 dogs were retrieved, for 20 hospitalizations and 28 KET IV injections over 3 years. KET IV boluses were used 12 times for RSE (9 generalized seizures, 3 focal seizures) and KET terminated the episode of RSE 12/12 times (100%); however, seizures recurred 4/12 times (33%) within ≤6 h of KET IV bolus. When used for CS apart from episodes of RSE, KET IV bolus was associated with termination of the CS episode only 4/14 times (29%). Only 4/28 (14%) KET IV boluses were associated with adverse effects imputable only to the use of KET. One dog experienced a short, self-limited seizure activity during administration of KET IV, which was most likely related to a pre-mature use of KET IV (i.e., before GABAergic resistance and NMDA receptor overexpression had taken place). This study indicates that KET 5 mg/kg IV bolus may be successful for the treatment of RSE in dogs.

## Introduction

The term cluster seizures (CS) refers to the onset of multiple generalized (tonic, clonic, or tonic–clonic) or focal seizures (with or without secondary generalization) within a 24-h period (1–4). Status epilepticus (SE) refers to the onset of continuous clinical or electroencephalographic seizure activity for 5 min or more, or to recurrent seizures/CS without recovery of normal mental and physical status between seizures (5–9). Convulsive SE is characterized by impairment of consciousness and prominent motor symptoms, usually in the form of sustained or interrupted bursts of abnormal muscle contraction, often bilateral (10). It is the most frequent presentation of SE in both humans and canine patients and can be associated with a high mortality rate in both species. Refractory status epilepticus (RSE) is defined as an onset of SE non-responsive to the standard emergency management of SE (11, 12), usually consisting in veterinary medicine of intravenous administration of benzodiazepine followed by another antiepileptic medication (AEM), such as levetiracetam (LEV) or phenobarbital (PB).

Status epilepticus and RSE are prolonged ictal events arising from failure of the mechanisms normally responsible for seizure termination. At the scale of a network of neurons, the main mechanisms of seizure termination include synaptic inhibition by presynaptic release of gamma amino-butyric acid (GABA), glutamate depletion, acidification of the intra- and extracellular space, and glial buffering of glutamate (13). GABAa receptors are chloride-conducting membrane channels with rapid opening after stimulation by GABA. During prolonged seizures in cases of SE/RSE, GABAa receptors are both desensitized and internalized, and the number of activated GABAa receptors on the postsynaptic membrane gradually decreases while the number of inactive GABAa receptors increases, leading to a decreased effect of GABAergic stimulation and ultimately resistance to benzodiazepines (14–17). Oppositely, the expression of *N*-methyl-D-aspartic (NMDA) receptors is upregulated during prolonged seizures in case of SE/RSE, leading to increased number and increased activity of the NMDA receptors. NMDA receptors are also the main receptor subtype involved in glutamatergic neurotransmission (18). Thus, prolonged seizures in cases of SE/RSE lead to a status of pharmacoresistance against the classical AEMs targeting the GABAergic system for seizure termination (e.g., diazepam, midazolam, phenobarbital, propofol) and potential neurotoxicity. While the GABAergic stimulation may appear as a pharmacological dead end during SE, NMDA receptor subunit migration toward the synaptic membrane (19), NMDA receptor activation during sustained neuronal stimulation, and possible glutamate excess due to failure of glial buffering present the NMDA receptors as an ideal therapeutic target.

Ketamine (KET), first synthetized by the American scientist Calvin Stevens in 1962 and first officially tested on human subjects in 1964 (inmates of the Jackson Prison in Michigan) (20), was developed as a derivative of phencyclidine (more commonly known as PCP or “angel dust”), after the withdrawal of the latter due to its undesired hallucinogenic effects. Ketamine is a schedule III substance on the United States Controlled Substances Act (USCSA) with an indication as a dissociative anesthetic. It is a fast-acting general anesthetic with significant analgesic activity and a relative lack of cardiopulmonary depressant effects. KET is also a potent NMDA receptor antagonist widely used in veterinary medicine.

To this day, administration of benzodiazepines, either at home ideally in the way of intranasal midazolam (MDZ) (21) or in the hospital intravenously, remains the mainstay of medical management of SE in veterinary medicine. For cases of sustained or repetitive seizure activity, a resistance to benzodiazepines can develop, leading to a lack of seizure termination despite classical therapy. Guidelines for second medication administration often include intravenous (IV) use of levetiracetam (LEV) or phenobarbital (PB) in dogs (22) and LEV, valproate, lacosamide, or phenytoin in humans. Third medication studies for those cases having failed previous therapeutic steps are sparse and usually involve the use of general anesthetic drugs such as propofol. Due to its NMDA glutamate receptor antagonist properties, KET has recently gained popularity as a therapeutic option for the treatment of SE/RSE in humans (23, 24). Multiple reports and studies document the use of KET for SE/RSE in human patients (12), either intravenously or orally, with frequent clinical success and seizure termination, specifically for cases of RSE refractory to GABAergic medications. In veterinary medicine, Serrano et al. reported in 2006 a case of RSE resistant to the previous use of rectal IV [bolus and constant rate infusion (CRI)] diazepam (DZ), oral PB, and IV propofol (CRI) that was responsive to KET IV bolus, with termination of RSE documented on electroencephalography (EEG). More recently, Gioeni et al. (25) reported on the use of a bolus and CRI of KET + dexmedetomidine combination, along with controlled mild hypothermia, to terminate prolonged RSE in three dogs.

The alteration in synaptic GABA responsiveness does not take place only in cases of RSE but can occur in the early stages of seizure/SE, as it has been shown that, within minutes, changes in GABAa receptor postsynaptic level and pharmacological status can develop (26), along with pharmacoresistance to certain anticonvulsants, including benzodiazepines (15, 27). CS have been shown to be associated with a significantly higher risk of developing SE/RSE in human patients than non-clustered seizures (2, 28). It was suggested that the mechanism leading to refractoriness in CS outlasts the immediate postictal phase (29) and that failure of seizure termination mechanisms, possibly loss of GABA-mediated inhibition, is the common pathophysiology to both CS and SE/RSE, with the former indicating a pharmacological pre-disposition to the latter (28, 30).

We have been using KET IV bolus in our hospital for cases of prolonged SE/RSE and CS with history of prolonged seizure activity despite benzodiazepine treatment prior to and/or during hospitalization. This use is based on the premise that KET, the most readily available intravenous NMDA antagonist in veterinary medicine, could help terminate SE and RSE in canine patients and may have a role in the management of hospitalized patients for prolonged and/or repetitive seizure activity. This retrospective study reflects our experience and observations. To the authors' knowledge, this is the first study documenting the use of KET IV bolus without following CRI for the treatment of CS/SE/RSE in a clinical setting in a cohort of client-owned pets.

## Materials and Methods

A search was made in the database of our hospital (ImproMed®, Henry Schein Veterinary Solutions, WI) using the terms “ketamine” and “seizure(s)” from January 2015 until January 2018. From that search, all the canine cases in which KET was used intravenously under supervision (direct, by phone, or consulting for another department) of either two of the authors (PR, AB) at Long Island Veterinary Specialists to treat seizures (CS, SE, and RSE) were recorded.

For this study the following definitions were used:

- Generalized tonic–clonic seizure: characterized by loss of consciousness and sustained or repetitive contraction of all muscles resulting in opisthotonus with clinical signs indicating involvement of both cerebral hemispheres during the ictal phase
- Focal seizure: clinical signs during the ictal phase indicative of involvement of only one or part of one cerebral hemisphere, with motor (e.g., facial twitching), autonomic (e.g., ptyalism), and/or behavioral components
- CS: ≥2 seizures within a 24-h period (regardless of the type of seizure)
- SE: uninterrupted clinical seizure activity for >5 min
- CS–SE: repetitive seizure activity (CS) with clinically identified and interrupted ictal phase but without recovery of normal consciousness (for >5 min)
- RSE: onset of SE refractory to in-hospital management with IV administration of at least two antiepileptic drugs, including a benzodiazepine
- Idiopathic epilepsy: epilepsy with proven or suspected genetic background, or epilepsy of unknown cause with no evidence of structural epilepsy
- Structural epilepsy: epilepsy caused by identified cerebral pathology
- Reactive seizure: seizure occurring as a natural response from the normal brain to a transient disturbance in function (metabolic or toxic in nature)

The medical records of all patients having received KET IV for seizure treatment at least once were reviewed by one of the authors (PR). All the patients included presented for seizures, with either CS, CS–SE, SE, or RSE. All the patients presented to the Emergency Department for repeated and/or continuous seizure activity, or due to owner's concern of lack of recovery of normal mental status after/between seizures (for cases of idiopathic epilepsy with regular CS episodes, usually managed at home per owner). All patients had an IV catheter placed in the cephalic vein of a thoracic limb or saphenous vein of a pelvic limb on admission. All patients received at least one IV dose of DZ at ≥0.5 mg/kg IV at admission when presented actively seizing (± other AEMs), or when seizure was detected during hospitalization afterwards and when feasible (in our hospital, each patient admitted for seizures is housed in the ICU with a loaded syringe with DZ at 0.5–1 mg/kg protected from light on the cage door). Each patient's daily AEM regimen at the time of admission to the hospital was maintained or increased during hospitalization. For each patient, the episode of CS, CS–SE, SE, or RSE for which KET was used intravenously was considered, and all available information pertaining to this hospitalization was reviewed, from admission to hospital (sometimes several days before the use of KET) to discharge time (sometimes several days after the last use of KET).

The following information was obtained from medical records for each patient: signalment (age, sex, breed), weight, presumptive or definitive diagnosis for the underlying cause of the seizure disorder [idiopathic epilepsy, structural epilepsy, reactive seizures, unknown cause—as defined earlier in the text and according to (31)], type of seizure(s) at presentation (e.g., focal vs. generalized), type of episode (i.e., CS vs. CS–SE vs. SE vs. RSE vs. combination) with approximate duration of seizure(s) prior to pharmaceutical intervention (when available), daily AEM(s) used for regular seizure management at time of hospitalization, AEM(s) with dosage and route of administration used prior to or in conjunction with KET for the control of the considered episode of CS/CS–SE/RSE, dosage and protocol used for KET administration, any adverse effect observed after KET, diagnostics performed at the time of hospitalization, outcome of KET on the episode of CS/CS–SE/SE/RSE, and outcome of the hospitalization. This information is presented in Supplementary Table A.

If KET was given ≤5 min of other AEM(s), the administration of KET was considered to be simultaneous to other AEM(s) administration. KET was considered administered solely if given >5 min from other AEM(s). KET was considered to have terminated seizure activity if cessation occurred <5 min from KET IV administration, and it was recorded to be either administered solely or simultaneous to other AEM(s). For example, if DZ IV injection did not stop seizure activity with KET IV administered >5 min after DZ, and cessation of seizure activity occurred <5 min after KET IV, KET was considered to terminate this seizure event solely.

## Results

A total of 15 dogs were retrieved, for a total of 20 hospitalizations and 28 IV KET boluses administered (see Supplementary Table A). Some cases presented a combination of several types of seizures during one hospitalization, for a breakdown of onsets of CS/CS–SE/SE/RSE as follows: *CS* (11 cases for 13 hospitalizations), *CS–SE* (8 cases for 10 hospitalizations), *SE* (7 cases for 7 hospitalizations), and *RSE* (10 cases for 11 hospitalizations). All the hospitalizations in which RSE was observed also included an episode of CS or CS–SE.

The different types of onsets/episodes of seizures treated during hospitalization and KET IV injections were recorded as follows (not limited to 1 per patient/hospitalization, as certain patients developed several of the specified patterns in 1 hospitalization and/or received KET IV several times, *e.g., case 3B presented for CS–SE and had an episode of SE in which ketamine was used*): *RSE of generalized seizure*: 7 patients/8 hospitalizations/9 injections, *RSE of focal seizure*: 3 patients/3 hospitalizations/3 injections, *SE*: 7 patients/7 hospitalizations/2 injections, *CS*: 11 patients/13 onsets/4 injections, and *CS–SE*: 8 patients/10 hospitalizations/10 injections.

Three patients received IV KET at two separate hospitalizations and one patient received it at three separate hospitalizations. Out of these 20 hospitalizations for seizures, KET was used as an IV bolus 28 times (6 patients received 2 boluses during 1 hospitalization, 1 patient received 2 boluses at each of its 2 hospitalizations). All boluses were administered at 5 mg/kg IV, except in 2/28 administrations: KET was administered once at 3 mg/kg IV and once at 6 mg/kg IV. Both doses are believed to be due to discrepancies in weight recording of the patients or miscalculation at the time of injection, as the dose intended was 5 mg/kg IV. The exact duration of the seizure activity (e.g., in case of SE/RSE) could not be accurately specified but rather estimated in most cases due to the limited temporal specificity obtained from the retrospective review of medical records, but all cases of RSE presented with uninterrupted clinical seizure activity for >15 min at a minimum, with at least 5 onsets of RSE (in 4 different patients) lasting for >30 min (up to several hours) prior to termination.

The underlying etiology of the seizure disorder for each KET administration was as follows: idiopathic epilepsy (18 injections of ketamine), reactive seizures due to metabolic disorder (hepatic encephalopathy) (4), structural epilepsy (3), and unknown cause (3).

Four patients had never been treated with any AEMs at the time of presentation. All these 4 cases presented for their first onset of seizure known to the owners. All other 11 patients were already on at least 1 AEM at the time of presentation with 5 patients (representing 8 hospitalizations) already on multiple daily AEMs at the time of onset of CS, CS–SE, SE, or RSE. Out of these 5 patients (representing 8 hospitalizations), 4 patients (representing 7 hospitalizations) were diagnosed with idiopathic epilepsy prior to the onset considered and would fit the classical definition of refractory epilepsy (poor control of seizure activity despite daily administration of at least 2 AEMs within their therapeutic range).

**Ketamine Was Used Intravenously for Different Onsets of Seizures With Results as Follows**

- *RSE of focal seizures*: 3 times: KET terminated RSE of focal seizures 3/3 times (used solely twice, with LEV 60 mg/kg IV + DZ 0.5 mg/kg IV once).
- *RSE of generalized seizures*: 9 times: KET terminated RSE of generalized seizures 9/9 times (used solely 8 times, with DZ 0.5 mg/kg IV once).
- *SE*: 2 times: KET terminated SE 2/2 times (used solely twice: once for focal seizure, once for generalized seizure).
- *CS–SE*: 10 times:
  - KET terminated the CS–SE with no seizure reported afterwards until discharge from the hospital 3/10 times (used solely twice, with DZ 0.5 mg/kg IV + LEV 60 mg/kg IV + clorazepate 1.25 mg/kg PO once).
  - KET failed to terminate the CS–SE but was followed by a decrease of seizure frequency within the cluster 7/10 times (used solely twice, with LEV 75 mg/kg IV once, with PB 1.67 mg/kg IV once, with DZ 0.5 mg/kg IV once, with DZ 0.5 mg/kg IV + PB IV + LEV IV twice).
- *CS*: 4 times:
  - KET terminated the CS with no more seizure afterwards until discharge from the hospital only once (used with LEV 150 mg/kg IV + PB 5 mg/kg IV).
  - KET failed to terminate the CS but was followed by a decrease of seizure frequency within the cluster twice (used solely twice).
  - KET was associated with seizure activity during KET IV administration once (used solely).

The results regarding the type of seizure event treated (i.e., RSE, SE, CS–SE, or CS) and the outcome of KET IV bolus are summarized in Table 1.

**Table 1:** Use of ketamine IV bolus by type of seizure event and associated outcome.

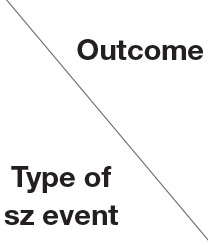	**KET terminated the sz event**	**KET decreased sz activity but failed to terminate the event**	**KET worsened sz activity**	**Total**
RSE SE CS–SE CS Total	12^a^ 2 3 1 18	0 0 7^b^ 2 9	0 0 0 1 1	12 2 10 4 28

*CS, cluster seizure; KET, ketamine; RSE, refractory status epilepticus; SE, status epilepticus; sz, seizure*.

a *One of the bolus administered was at 6 mg/kg*.

b *One of the bolus administered was at 3 mg/kg*.

The results regarding the administration of KET with or without other AEMs IV and the outcome are summarized in Table 2.

**Table 2:** Use of ketamine IV with or without other drugs with associated outcome.

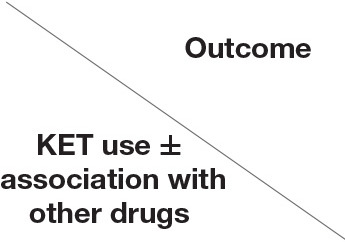	**KET terminated the sz event**	**KET decreased sz activity but failed to terminate the event**	**KET worsened sz activity**	**Total**
KET only KET + DZ KET + LEV/PB (or both) KET + LEV/PB (or both) Total	14^a^ 1 1 2 18	2^b^ 3 2 2 9	1 0 0 0 1	17 4 3 4 28

*AEM, antiepileptic medication; DZ, diazepam; KET, ketamine; LEV, levetiracetam; PB, phenobarbital; sz, seizure*.

a *One of the bolus administered was at 6 mg/kg*.

b *One of the bolus administered was at 3 mg/kg*.

In the majority of cases, there were no obvious adverse effects reported on the use of KET IV.

In 23/28 administrations, the sole impact of KET IV on the patient's mental status could not confidently be evaluated, as the patients were already showing significantly altered mental status at the time of KET IV administration, as a consequence of either the episode of CS/CS–SE/SE/RSE considered, the other AEMs previously or concurrently administered (e.g., IV DZ or PB), or of the underlying intracranial pathology. In the remaining 5 administrations, KET IV was followed by no obvious/documented impact on mentation in 2 administrations, inappropriate/dysphoric mental status observed shortly after KET IV (required sedation with DZ IV ± other drugs) in 2 administrations, and 2 short self-limiting seizures were observed following 1 administration. It is noteworthy that at least 3 patients with CS or CS–SE were reported to be able to take oral medications and walk <15 min after KET IV bolus (could only be ascertained in 3 dogs/3 hospitalizations due to the retrospective nature of medical records review).

Blood pressure (BP) measurements obtained after KET IV administration were either unavailable or not considered representative/accurate for comparison (e.g., if BP measurement post-KET was >6 h after KET IV injection) for 20/28 administrations. For the remaining 8 administrations of KET IV bolus, BP measurements showed at least one measurement of BP ≥160 mmHg after KET IV in 3/8 administrations (including 1 patient with elevated BP prior to KET IV which normalized within 6 h of KET) and were within the normal range for 5/8 administrations (including 1 patient with elevated BP prior to KET IV).

No gastrointestinal side effects (e.g., vomiting, diarrhea) were recorded after KET IV in any of the cases. Temporary excessive ptyalism was documented following KET IV in 1/28 administrations.

In summary, KET IV administrations were documented to be associated with adverse effects imputed to KET in only 6/28 injections: 1 patient who received KET IV during a CS event but not at a time of ongoing ictal activity exhibited 2 short self-limiting seizures during KET IV administration; 2 patients exhibited inappropriate/dysphoric mental status; 1 patient exhibited temporary excessive ptyalism; and 2 patients had a temporary elevated BP.

Regarding the outcome of hospitalization, 16/20 hospitalizations resulted in discharge from the hospital (with 1 case coming back within hours for persistent seizure activity) and 4/20 hospitalizations resulted in euthanasia, despite cessation of seizure activity in the hospital (all 4 patients euthanized presented at least one episode of RSE). Euthanasia was requested per owner due to a combination of prognosis associated with intracranial disease (3/4 dogs euthanized were diagnosed with structural or presumptive structural epilepsy), psychological impact of the episode of RSE on the owners, and perceived poor quality of life.

## Discussion

SE is a clinical entity frequently encountered in veterinary medicine, with ~59% of dogs with epilepsy of any kind experiencing at least one episode of SE in their lifetime (32). It is a neurologic emergency with a high mortality rate in both humans (up to ~20%) (9, 33–36) and dogs (estimated ~25% per certain authors) (37). Monteiro et al. (38) reported in a study of 407 dogs that 47% of dogs with idiopathic epilepsy (IE) experienced CS at least once in their epilepsy history. Border Collies with confirmed IE are reported to have occurrences of CS in 94% and SE in 53% of the cases (39). Australian Shepherds with IE diagnosed before 5 years of age are reported to suffer from CS and/or SE in 80% of the cases, with almost half of the population suffering from both (40). The impact of these episodes on the overall management of epilepsy is major, as the repeated cost of hospitalization in an intensive care unit (ICU), emotional burden on owners and their family, and potential comorbidities associated with SE (e.g., aspiration pneumonia) are likely to lead to euthanasia. RSE, despite being clinically encountered relatively frequently, is sparsely documented in veterinary medicine. Although an accurate estimation of the morbidity and mortality rate of RSE in dogs is difficult to establish, it has been associated with a mortality rate as high as 23–61% and a relapse rate of up to 90% in human survivors (6, 12, 41–43). This is further complicated in veterinary medicine by the frequent outcome of euthanasia solicited by caretakers in dogs surviving an onset of RSE, often for financial or perceived quality-of-life (QOL) reasons, as found in this cohort with 4/10 patients who developed RSE being euthanized, despite termination of seizure activity in the hospital. The relapse rate was also high in this cohort as, out of the 6 remaining patients who developed RSE and were not euthanized before discharge, 4 patients experienced at least one other episode of RSE to the authors' knowledge (included in the study or not), leaving only 2/10 patients who experienced RSE not relapsing nor being euthanized. There was also a subgroup of recidivists identified, with 3 dogs being responsible for 6/20 hospitalizations and 9/28 injections of KET IV; all 3 of these dogs were diagnosed with idiopathic epilepsy. These findings are in favor of a possible pharmacological predisposition to RSE for a subset of the population of epileptics in veterinary patients, as discussed previously in humans.

NMDA receptors are non-specific cation channels containing the NMDA and phencyclidine (PCP) binding sites and are also the main receptor subtype involved in glutamatergic neurotransmission (18). Although its complete mechanism of action is not yet fully understood (44), KET, a non-competitive antagonist for NMDA receptors, binds to the PCP site inside of the ion channel of the NMDA receptor. This results in blockade of the intracellular flow of Ca^2+^ and Na^+^ that normally occurs after activation of the NMDA receptor, following the removal of the Mg^2+^ from the inner side of the ion channel under the combined effects of glutamate and glycine (45). This cellular mechanism of action is associated with reduced epileptiform burst discharges and after-potentials, resulting in inhibition of excitation conduction and anticonvulsive role (46), and may explain the efficacy of KET for SE/RSE in a broad range of seizure etiology.

The route of administration and dose used in this study were decided based on several factors. KET readily penetrates the blood–brain barrier due to its relatively low plasma protein binding rate (37–53% in cats, 53% in dogs) and fat solubility, and maximum plasma concentration is reached within 1–5 min after IV administration (12, 47, 48) making it a fast-acting drug when used IV, ideal in the setting of CS/CS–SE/SE/RSE. This fast to immediate action has been supported by other studies (49–51) and is one of the reasons why the 5-min time frame was used for KET IV injection to be considered successful at terminating ictal activity and separate from other IV injections in this retrospective study. Five minutes has been used as the time limit to consider treatment successful in previous veterinary studies on canine SE (21, 52). Most drugs injected IV within the context of SE, including DZ, are expected to be successful within that period (9, 53, 54), and it is also a frequent time frame for emergency clinicians to reach for another therapeutic option if the last one was unsuccessful. Due to the retrospective nature of this study, it was also a time frame that could be reasonably verified in medical records. The dose of 5 mg/kg was arbitrarily chosen and has been previously reported by other authors in veterinary patients (9, 55). The use of KET CRI has been documented in both human and veterinary patients (55–57) but was not evaluated in this study.

Perhaps the most interesting finding of this study is the fact that KET 5 mg/kg IV, as sole agent (9 injections) or in combination with other AEMs such as DZ and/or LEV (3 injections), resulted in the termination of RSE in 12/12 episodes of RSE (100%) and 2/2 episodes of prolonged SE, where other AEMs or anesthetic agents classically used (e.g., DZ, PB, LEV, propofol) had previously failed. One of the authors (PR) has also used the same treatment successfully in a case of feline RSE due to structural epilepsy. This efficacy of KET to terminate RSE has been documented in several human studies, with Synowiec et al. (58) reporting a similar success rate in 11 patients with RSE, all successfully terminated with KET. Review of the human literature also confirms the effectiveness of KET in cases of RSE, however with more mitigated results with studies documenting termination of RSE in 56.5–100% of the cases (12, 56, 57, 59). It is likely that larger/future veterinary studies may not report such a high efficacy of KET for RSE as the one reported in this cohort; however, a few factors may explain why such efficacy can be observed and should be kept in mind when dealing with cases of RSE. First, and as discussed in the Introduction, prolonged ictal state is associated with downregulation of GABAa receptors and upregulation of NMDA receptors, leading to a state of pharmacoresistance to benzodiazepines and other AEMs. In order to be effective at terminating the ictal state, KET requires this upregulation of NMDA receptors to have occurred for its site of action to be available. This has been documented in a murine study of SE, with PB efficacy decreasing as the time of SE increased, while KET administration was associated with an “all or nothing” response with 0/4 (0%) SE terminated when KET was administered after 15 min of seizures, but 4/4 (100%) SE terminated when KET was administered after 1 h of seizures (60). This phenomenon has also been observed clinically by previous authors in both humans and veterinary patients (12, 55, 61), and the rationale of the use of KET for RSE in a clinical setting could be summarized by the rule of thumb “the longer the seizure activity has been, the more GABAergic medications have failed, the more likely KET will terminate RSE.” Although an accurate determination of the time of seizures prior to presentation could not be made in most cases, the fact that KET was used for cases of RSE when several AEMs had already failed to stop seizure activity implies that most cases were treated after prolonged period of ictal stage and at a time when pharmacoresistance to GABAergic AEMs had developed and may explain the success rate observed for RSE in this cohort. It is interesting to note that the only patient who did not develop SE/RSE in this study is also the only patient who developed seizures during the administration of KET.

To the best of the authors' knowledge, it is unclear how long the refractory state to benzodiazepines may persist after seizure termination in cases of SE/RSE. The literature on changes in GABA receptor trafficking associated with SE is richer regarding the development of downregulation and refractory status, than on the recovery of normal state following seizure termination (62). Studies have shown that GABAa receptor trafficking and replenishment of the synaptic pool can occur through exocytosis of intracellular receptors, recapture/endocytosis of extracellular receptors, or by lateral diffusion along the cellular membrane plane of extrasynaptic receptors into the synaptic domain (62, 63). The first two mechanisms are relatively slow and are downregulated during prolonged seizure activity, while lateral diffusion of extrasynaptic GABAa receptors toward the synaptic domain was shown to be likely the main mechanism and to replenish functional GABAa receptors at inhibitory synapses within minutes in an experimental model (64). There is also rapid overexpression of certain GABA receptor subunits such as γ2 in the hippocampus, potentially compensating for the initial loss following SE (65, 66). It is interesting to note that, several hours after termination of RSE by KET IV, certain patients in this study exhibited recurrences of either RSE requiring further KET IV injection or seizures that were terminated successfully with DZ IV only. This may represent an alternance of periods of refractory and sensitive GABAergic pharmacological status throughout a CS/CS–SE episode in certain patients. As the authors are unaware of any studies documenting the duration of such refractory period following SE/RSE in a clinical setting, this may justify treating the first seizure after a period of refractory status with DZ rather than assuming that a refractory state is persisting.

The efficacy of KET IV in cases of CS/CS–SE is more difficult to determine in this study. Out of 14 KET injections to treat CS or CS–SE, only 4/14 (29%) resulted in the termination of the CS/CS–SE event, while 10/14 (71%) were associated with decreased seizure frequency within the CS/CS–SE but repeated seizure activity requiring further AEMs. Most of the patients treated also received multiple AEMs simultaneously to KET (7/14 injections of KET were with DZ ± other AEMs) or prior to KET, including oral medications with a potential delayed effect on seizure frequency. This simultaneous multiple therapy using AEMs with different target receptors and KET has been shown to be associated with higher efficacy, reduced neuronal injury, and improved therapeutic index compared with sequential multiple therapy or high-dose single therapy in murine models of SE (23, 24). A clinical study in dogs with CS or SE also showed synergistic effect of using LEV in addition to DZ compared with DZ only (67). This is most likely related to the changes in receptor trafficking involving both GABAergic and glutamatergic pathways involved in the development of pharmacoresistance and may constitute an area of interest for future studies. Another possible interest in simultaneous multiple therapy involving KET is to mitigate the side effects associated with potentially high doses of other AEMs classically used (e.g., KET could have a “vasopressor-sparing” effect considering the depressant action of PB on blood pressure, specifically when administered after DZ/MDZ).

RSE currently somewhat lacks an established, consensual definition in veterinary medicine. The International Veterinary Epilepsy Task Force (IVETF) published in 2015 a consensus report on epilepsy terminology in companion animals (31) but did not include RSE in the terminology suggested. RSE has been defined in veterinary medicine as “status epilepticus that does not respond to a benzodiazepine or PB” (i.e., not including the requirement of treating with at least two different AEMs) (9) and in human medicine as “recurrent seizure activity [or] ongoing seizures despite two appropriately selected and dosed antiepileptic drugs including a benzodiazepine” (i.e., two different definitions in the same text, the first one including cases of recurrent seizure activity hence not specifying the need for continuous seizure activity commonly associated with RSE) (11) or as “status epilepticus (SE) that cannot be resolved in terms of clinical manifestations or epileptiform discharges following the rational administration of anticonvulsants including a benzodiazepine” (12). The authors chose to adhere more strictly to a definition including prolonged seizure activity without cessation, despite the use of benzodiazepine IV and at least one other AEM IV at the time of the prolonged SE. All 12 cases but 1 of RSE included in this study received 2 or more DZ injections at ≥0.5 mg/kg IV and at least 1 other AEM IV prior to KET at the time of RSE (most commonly LEV 60 mg/kg IV and/or PB 5–16 mg/kg IV ± propofol). One dog received only 1 injection of DZ (and LEV IV as discussed above) prior to KET at the time of RSE (but had received DZ shortly before due to previous seizure activity). One of the cases treated fulfill the criteria for super-RSE, since it has been defined in humans as an RSE onset that does not terminate or does recur following continuous IV administration of anesthetic agents (e.g., propofol) for more than 24 h (12). The definitions of SE sometimes used in human medicine and suggested by the IVETF for veterinary medicine as “(a) >5 min of continuous epileptic seizures or (b) two or more discrete epileptic seizures between which there is incomplete recovery of consciousness (for generalized convulsive seizures)” (31) do not differentiate between continuous, uninterrupted seizure activity (a) and repetitive but interrupted seizure events (b). In a population of dogs on polypharmacy affecting consciousness and often presenting with abnormal mentation (e.g., many of the patients in this study were already on multiple AEMs with extra doses given and/or received benzodiazepines such as CZ, DZ, and MDZ prior to presentation), this would result in cases of CS being categorized as SE. By extension, if refractory to DZ + other AEM IV and no recovery of consciousness between seizures of a cluster, some could be considered in RSE while these dogs, in fact, did not present the continuous, uninterrupted seizure activity specific to prolonged SE/RSE. This is relevant clinically when treating this condition since, as discussed earlier in the text, prolonged ictal state (not merely recurrent seizures) is associated with and required for GABAa receptor downregulation and NMDA receptor upregulation, a pre-requisite for the successful termination of ictal status by NMDA antagonists such as KET. The definition of SE as strictly uninterrupted seizure activity [(a) only in the terminology suggested by the IVETF] and the use of the term CS–SE in this study [similar to (b) in the terminology for SE suggested by the IVETF] were justified to try and avoid “lumping” together two populations of patients who were likely not in the same pharmacological state (one having developed NMDA upregulation while the other has not) and in accordance with the IVETF statement that “our definitions should be seen as clinically operational” (31). The results obtained in this group with KET terminating the ictal event in all cases of prolonged, uninterrupted seizure activity [RSE 12/12 times (100%) and SE 2/2 times (100%)], but in only 4/14 of the CS and CS–SE (see Table 1), are the clinical reflection of this pharmacological difference.

As discussed previously, isolating the possible adverse effects of KET is difficult at best in the context of CS–SE/SE/RSE. It is, however, noteworthy that only 6/28 injections were associated with adverse effect imputable only to KET (see results). The dose used of 5 mg/kg bolus is not an anesthetic dose and did not require intubation or specific measures to protect airways in any cases in this study. The classically reported proconvulsant, psychotropic, hypertensive, and hypersalivation effects of KET were observed, respectively, in this study in only 1/28, 2/28, 2/28, and 1/28 injections. This is likely related to the specificity of treating at a time of prolonged seizure activity, as patients are often already mentally severely altered (as a result of seizure activity, intracranial pathology, and/or AEMs) and occasionally hypotensive (due to previous repetitive use of benzodiazepines and PB) or hypertensive (due to continuous ictal activity, which KET may normalize by terminating SE/RSE). Although historically reported to have an increasing effect on intracranial pressure (ICP) (47, 68, 69), a systematic evaluation of KET for non-traumatic neurological diseases (70) and a study on 58 human patients (56) reported no case of significant ICP elevation imputable to KET only. Subjectively, the authors have not yet observed significant adverse effects associated with KET 5 mg/kg IV bolus when used for prolonged seizure activity. KET has been reported to have potentially a proconvulsant effect in veterinary patients, with up to 20% of cats in one study and reports of dogs developing seizures after induction with KET IV (71–73). The reasons why KET may be proconvulsant for some patients are not completely understood, but a genetic pre-disposition may exist, since a phenotypic pre-disposition was described in the feline cohort with only all of the cats with prominent black or dark brown stripes on their hair coat developing seizures (71). As discussed earlier, one dog experienced short, self-limited seizure activity during administration of KET IV, which was most likely related to a “pre-mature” use of KET IV (i.e., before GABAergic resistance and NMDA receptor overexpression had taken place), since this patient was the only one to never develop CS–SE/SE/RSE in this study.

The downregulation of GABA and the upregulation of NMDA receptors are not the only reasons for pharmacoresistance to benzodiazepines and other drugs in cases of prolonged seizure activity. There is increasing evidence that blood–brain barrier dysfunction, neuroinflammation, and upregulation of blood–brain barrier efflux transporters such as P-glycoprotein occur in refractory stages of SE, leading to failure of the drugs to reach the target and possible pharmacoresistance (74–76). This opens the possibility for different modalities of treatment in the future, although it was not evaluated in our study.

Most of the limitations of this study are inherent to a retrospective study and directly related to the subject of the study: treatment of CS–SE/SE/RSE. This difficulty in interpreting heterogeneous data retrospectively was reported in a systematic review of NMDA receptor antagonists for the treatment of RSE in humans by Zeiler et al. (59), with limitations related to the small sample sizes in studies on RSE, the heterogeneity of AEMs used prior to KET, the timing of different AEMs used, and the duration of such medications. All these difficulties were encountered in this study, with also a broad range of underlying etiologies (idiopathic, structural, reactive, unknown) and AEMs used prior to KET use, rendering (as examples) impossible to isolate the effect of KET on patient's mentation in most cases and difficult to ascertain the sole impact of KET on cases of CS/CS–SE. Evidence-based medicine (EBM) aims at guiding the treatment/care for individual patients based on clinical expertise and educated use of the best available medical evidence (77), the latter being ideally in the form of double-blinded, randomized, controlled prospective clinical trials or large meta-analysis. To the best of the authors' knowledge, no clinical controlled prospective studies have been published regarding the use of KET for the treatment of RSE, whether in human or veterinary medicine. Considering the high mortality and morbidity associated with RSE, and for obvious ethical reasons, it is difficult to establish randomized controlled clinical trials in such patients. The “gold standard” might then be in the form of future larger prospective studies, with the intent to establish a standardized protocol regarding the best administration of KET (in terms of timing, dosage, use of boluses vs. CRI).

In conclusion, review of laboratory models and results of human studies, taken collectively, suggest that ketamine seems primarily suitable and safe for the treatment of prolonged and uninterrupted seizure activity in cases of SE/RSE. The role of NMDA antagonists, KET or others such as dextromethorphan (78), in the pharmacological arsenal deployed in cases of *CS/CS–SE/SE/RSE* warrants further investigation and prospective studies, specifically regarding ideal protocol for administration (e.g., dosage, use of CRI) and proper case selection. In the meantime, our results suggest that KET 5 mg/kg IV bolus, solely or in combination with other AEM(s), may be useful in the treatment of canine RSE. The authors also recommend that the use of KET IV be limited to cases of SE/RSE that exhibit prolonged, uninterrupted seizure activity and resistance to benzodiazepines.

## Data Availability Statement

The original contributions generated in the study are included in the article/Supplementary Material.

## Ethics Statement

Ethical review and approval was not required for the animal study because this is a retrospective clinical study. Written informed consent was obtained from the owners for the participation of their animals in this study.

## Author Contributions

PR contributed initial hypothesis that ketamine would terminate refractory status epilepticus, conception and design of the study, treatment of clinical cases, collection of the data, and redaction of entire manuscript. AB contributed treatment of clinical cases and redaction of manuscript. CWD contributed redaction of manuscript. All authors contributed to manuscript revision, read and approved the submitted version.

## Conflict of Interest

The authors declare that the research was conducted in the absence of any commercial or financial relationships that could be construed as a potential conflict of interest.
